# Thyroid Hormone Replacement Dose Is Not Associated with Anti-TPO and Anti-TG Antibody Titers in Hashimoto’s Disease

**DOI:** 10.3390/jcm15030970

**Published:** 2026-01-25

**Authors:** Małgorzata Szczuko, Olimpia Szmigiel, Urszula Szczuko, Leon Rudak, Karolina Wrońska, Lidia Kwiatkowska, Małgorzata Tomasik, Anhelli Syrenicz, Jakub Pobłocki

**Affiliations:** 1 Department of Bromatology and Nutritional Diagnostics, Pomeranian Medical University in Szczecin, 71-460 Szczecin, Poland74985@student.pum.edu.pl (K.W.); jakub.poblocki@pum.edu.pl (J.P.); 2 Department of Interdisciplinary Dentistry, Faculty of Medicine and Dentistry, Pomeranian Medical University in Szczecin, 70-111 Szczecin, Poland; malgorzata.tomasik@pum.edu.pl; 3 Department of Endocrinology, Metabolic Diseases and Internal Diseases, Pomeranian Medical University in Szczecin, 70-252 Szczecin, Poland; anhelli.syrenicz@pum.edu.pl

**Keywords:** Hashimoto’s thyroiditis, anti-TPO, anti-TG, L-thyroxine, levothyroxine

## Abstract

**Background**: Hashimoto’s thyroiditis (HT) is the result of a complex interplay between genetic, environmental, and epigenetic factors. The role of cellular and humoral immunity in the pathogenesis of the disease is well-established. Inflammatory infiltration of T and B lymphocytes is a key feature identified on ultrasound examination. The lack of data on the effect of L-thyroxine (LT-4) doses on the level of anti-TPO and anti-TG antibodies in Hashimoto’s thyroiditis and the relationship with anthropometric measurements resulted in the desire to fill this niche. **Methods**: A total of 70 Caucasian patients diagnosed with Hashimoto’s thyroiditis within the past two years were examined. The participants were divided into three groups based on their L-thyroxine dosage (≤50, 50–100, >100 μg). **Results**: The results revealed no correlation between the dosage of L-thyroxine and anthropometric measurements (age, height, body weight, and body fat content). No correlation was identified between the levels of anti-TPO and anti-TG and the dose of L-thyroxine in patients with Hashimoto’s thyroiditis. **Conclusions**: The mechanism regulating the levels of anti-TPO and anti-TG appears to be associated with a more advanced thyroid inflammation and disease process. Long-term observation of patients would be advisable. We present evidence of no effect of hormone dose on antibody levels in Hashimoto’s thyroiditis. Regardless of disease severity, immune regulation remains outside the scope of hormonal regulation.

## 1. Introduction

The pathogenesis of Hashimoto’s thyroiditis (HT) is determined by immune dysfunction from the accumulation of autoreactive lymphocytes and the resultant loss of immune tolerance to autologous tissue, which ultimately leads to the destruction of the thyroid gland and the development of hypothyroidism [1]. Although the exact etiology of the disease is not yet fully understood, Hashimoto’s thyroiditis is attributed to the interaction of genetic, environmental, and epigenetic influences [2]. The role of inflammation in the pathogenesis of Hashimoto’s thyroiditis is well-documented, characterized by an inflammatory infiltrate of T and B lymphocytes [2]. Thyroid peroxidase antibodies (anti-TPO) and thyroglobulin antibodies (anti-TG) are produced by B lymphocytes in combination and have been shown to cause greater damage to thyroid tissue than T-cell or cytokine-mediated apoptosis [3]. The thyroglobulin gene may be susceptible to autoimmune thyroiditis by encoding thyroglobulin variants with different immunogenicity [4]. Consequently, the thyroid gland may represent a significant site for thyroid antibody secretion, as evidenced by the observed decrease in serum thyroid antibodies following surgical intervention and during anti-thyroid drug administration. Light microscopic analysis revealed the accumulation of T/cytotoxic suppressor cells in areas of thyroid follicle destruction, which were characterized by the presence of connective tissue fibers and fibroblasts [5]. T helper type 2 cells (Th2) have been shown to overstimulate and promote B lymphocytes and plasma cells, which, in turn, produce antibodies against thyroid antigens and cause thyroiditis. In contrast, Th1 and Th2 lymphocytes have been observed to produce interferon (IFN-gamma) and interleukin 4 (IL-4), respectively. Shen et al. [6] found that IFN-gamma and IL-4 gene polymorphisms with high IFN-gamma production and low IL-4 production were more common in patients with severe thyroiditis. Polymorphisms in the MAGI3 (membrane-bound guanylate kinase) gene are associated with an increased risk of hypothyroidism in individuals with high levels of autoantibodies against thyroglobulin [7]. Autoantibodies (anti-TG and anti-TPO) are typically associated with disease activity. A substantial body of research has demonstrated that the serum concentrations of thyroid peroxidase antibodies in patients with Hashimoto’s thyroiditis decline following L-thyroxine (LT-4) treatment [8]. Previous scientific reports have indicated an association between autoantibody titers, levothyroxine doses, and clinical symptoms in patients [9]. Antibody titers are considered a marker of disease severity rather than a direct determinant of levothyroxine dosage. Altun et al. [10] conducted a longitudinal study on 101 patients with hypothyroidism over a period of approximately 60 months. A key objective of this study was to ascertain whether L-thyroxine replacement therapy impacts the progression of the disease. In the group of patients treated with LT4 during the 5-year follow-up period, the anti-TG level remained unchanged; however, the anti-TPO level demonstrated a significant decrease during the follow-up period (*p* < 0.001). Population studies have shown that thyroid antibody levels increase with age, especially in women [11]. Previous studies have confirmed the occurrence of abnormalities in anthropometric parameters in individuals with HT compared to healthy individuals. Higher body weight, body mass index (BMI), fat mass, and Waist–Hip Ratio (WHR) were found [12]. Scientific reports also suggest an association between leptin, hypothyroidism, and a vicious circle of excess weight in people with HT. A study published in 2023 confirmed a significant correlation between leptin and BMI in patients with HT (r = 0.533, *p* < 0.001). Increased leptin levels are associated with activation of the hypothalamic–pituitary axis and increased thyroid-stimulating hormone (TSH) production, affecting the body’s energy expenditure [13]. Compared to patients of normal weight, obese patients had lower levels of free triiodothyronine (fT3) and free thyroxine (fT4) (*p* < 0.01), a higher incidence of hypothyroidism (*p* < 0.05), and a higher incidence of anti-thyroid antibodies (*p* < 0.05). As a marker of Autoimmune Thyroid Disease (AITD), thyroid peroxidase antibodies were more common in the obese group (*p* < 0.01) [14]. Although the relationship between anthropometric parameters and anti-thyroid antibodies has been confirmed, there is no clear explanation for the mechanisms behind this problem. The literature indicates that obesity, as a chronic low-grade inflammatory condition, stimulates excessive immune system activity, and hormones such as leptin may modify the immune response by promoting Th1 lymphocyte synthesis and inhibiting T regulatory cells (Treg), thereby increasing the production of anti-thyroid antibodies [15]. The primary hypothesis of this study is that increasing the dose of levothyroxine affects the level of anti-thyroid antibodies in patients diagnosed with HT. Furthermore, we cautiously hypothesize that the efficacy of antibody reduction might be related to an individual patient’s anthropometric parameters. The objective of this study is to examine the impact of levothyroxine dosage on antibody levels and anthropometric measurements among patients with Hashimoto’s thyroiditis.

## 2. Material and Methods

### 2.1. Characteristics of the Study Group

Caucasian women diagnosed with Hashimoto’s thyroiditis were eligible for inclusion in the study, and characteristics are provided in Table 1. The mean age of the 70 women who participated in the study was 36.68 years ± 8.34 years.

An ultrasound examination and measurement of hormone and antibody levels were performed. Qualification for this study was based on an ultrasound image typical for cAITD (ALOKA Prosound Alpha-7 device, linear probe UST-5411 4.4–13.3 MHz, Tokyo, Japan) and an elevated level of anti-TPO antibodies (>34 IU/mL) and/or anti-TG (>115 IU/mL) in blood serum. The disease was diagnosed within the last two years (new diagnosis or treatment initiated within the previous two years). Exclusion criteria encompassed malabsorption syndromes, bariatric surgery, intestinal or gastric resection, partial and total thyroidectomy, J-131 treatment, Graves’ disease, pregnancy, diabetes, arterial hypertension, coronary artery disease, severe systemic diseases, use of glucocorticosteroids, nonsteroidal anti-inflammatory drugs (referring to chronic use) and immunosuppressive drugs within the last six months, use of drugs directly affecting the function of the thyroid axis (except levothyroxine), and statin therapy. Immunoglobulin A (IgA) deficiency and coeliac disease were also excluded.

The most frequently reported clinical symptoms were weight gain, cold extremities, dry skin, thyroid enlargement on ultrasound, and swelling of the anterior neck. Less frequently reported symptoms included muscle pain, hair loss, sleep and concentration disturbances, joint pain, and mood disorders, including depression and anxiety. It was observed that all patients were either anti-TPO-positive or both anti-TPO- and anti-TG-positive. The women were divided into three groups based on the dose of L-thyroxine taken. The first group, comprising 30 participants, received a dose of levothyroxine up to ≤50 μg. The second group, with 30 members, received a dose ranging from 50 to 100 μg, and the third group, receiving a dose >100 μg, consisted of 10 women. This division determined the number of participants in the subgroups, allowing for statistical analysis.

### 2.2. Equipment and Biochemical Measurements

Anthropometric indices such as age (in years), height (in centimeters), body weight (in kilograms), and body fat percentage (in percentage form) were measured using established methods. The body composition of the subjects was assessed using the DXA (Dual Energy X-ray Absorptiometry) method, which employs a dual-beam X-ray absorptiometry system (GE Lunar Prodigy Advance, Madison, WI, USA) and utilizes the manufacturer’s proprietary software (CoreScan). Thyroid ultrasound was also performed using an ALOKA Prosound Alpha-7 device (Hitachi Healthcare, Tokyo, Japan) with a UST-5411 linear transducer (4.4–13.3 MHz, Hitachi Healthcare, Tokyo, Japan).

Blood biochemical tests, including the level of anti-TPO, anti-TG antibodies, and the levels of hormones TSH, fT3, and fT4, were performed using the electrochemiluminescence method (ECLIA) (Cobas 6000 module 601, Roche, Meylan, France) in an accredited hospital laboratory.

### 2.3. Statistical Analysis

In order to conduct the statistical analysis of the above-described study, we used STATISTICA v.13.3 software (StatSoft, Krakow, Poland). Baseline patient characteristics were summarized descriptively, and comparisons between independent groups were performed using one-way analysis of variance (ANOVA) or the Kruskal–Wallis test, depending on data distribution. For repeated measurements within the same subjects, appropriate tests for dependent samples were applied. Subsequently, associations between the administered L-thyroxine dose and anthropometric as well as biochemical parameters were assessed using Spearman’s rank correlation coefficient. Spearman’s correlation was selected to evaluate relationships between ordinal and quantitative variables that did not follow a normal distribution. Statistical significance was defined as *p* < 0.05.

## 3. Results

The mean height was 166.19 cm ± 5.74 cm, the mean body weight was 71.54 kg ± 13.03 kg, the mean percentage of body fat was 35.52% ± 6.75%, and the mean anti-TPO value was 232.83 IU/mL ± 276.98 IU/mL. The mean anti-TG value was found to be 335.85 IU/mL ± 615.92 IU/mL, the mean TSH value was 2.91 μIU/mL ± 2.35 μIU/mL, the mean fT3 value was 2.97 pg/mL ± 0.43 pg/mL, the mean fT4 value was 1.29 ng/dL ± 0.20 ng/dL, and the mean L-thyroxine dose was 71.16 μg ± 36.15 μg. Patients with excessively high body fat content averaged 35.52%. The study group’s characteristics are detailed in Table 1.

No significant correlations were observed between levothyroxine dose and selected anthropometric parameters in the study group. A summary of the correlations obtained is presented in Table 2.

Dividing the studied group of patients into three subgroups (I: ≤50 μg L-thyroxine; II: 50–100 μg L-thyroxine; III: >100 μg L-thyroxine) did not lead to the identification of any statistically significant between-group differences for anti-TPO, anti-TG, fT3, or fT4. Only the comparison of TSH between subgroups I and III had a *p*-value equal to 0.026, indicating a significant difference. (Table 3). In biochemical tests of the examined women taking L-thyroxine in group 1, the mean anti-TPO value = 231.17 ± 213.32 IU/mL, anti-TG = 309.99 ± 700.77 IU/mL, TSH = 3.10 ± 1.47 μIU/mL, fT3 = 3.03 ± 0.39 pg/mL, and fT4 = 1.26 ± 1.18 ng/dL. In group 2, the mean anti-TPO value = 170.93 ± 205.57 IU/mL, anti-TG = 393.24 ± 612.95 IU/mL, TSH = 3.12 ± 3.09 μIU/mL, fT3 = 2.93 ± 0.46 pg/mL, and fT4 = 1.32 ± 0.21 ng/dL. In the last study group, the mean anti-TPO value = 423.51 ± 475.23 IU/mL, anti-TG = 247.00 ± 187.72 IU/mL, TSH = 1.68 ± 1.30 μIU/mL, fT3 = 2.92 ± 0.42 pg/mL, and fT4 = 1.34 ± 0.19 ng/dL. The average anti-TPO level was lowest in group II (50–100 μg L-thyroxine) and highest in group III (>100 μg L-thyroxine). Conversely, the average anti-TG level was highest in group II (50–100 μg L-thyroxine) and lowest in group III (>100 μg L-thyroxine). However, these results were not statistically significant. A detailed analysis of the mean biochemical parameters and differences in the subgroups studied is presented in Table 3.

**Figure 1:**
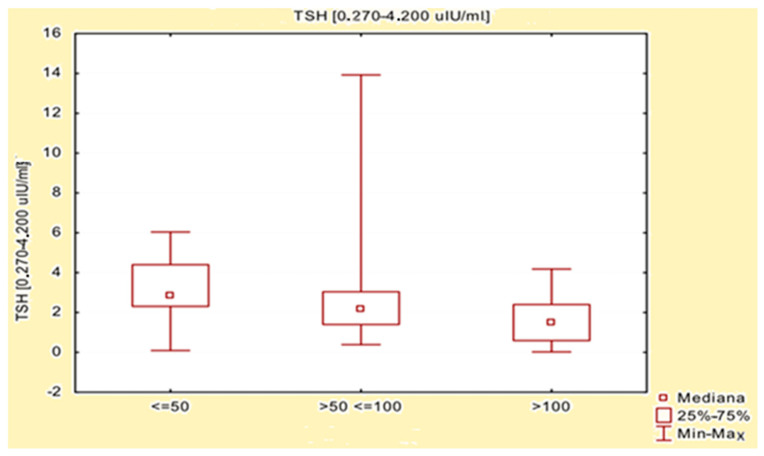
Comparison of TSH levels depending on the levothyroxine dose taken.

The effect of L-thyroxine dose on biochemical parameters (anti-TPO, anti-TG, TSH, fT3, and fT4) was evaluated using the Kruskal–Wallis test, and the results are presented in Table 4. Significant differences were observed in the groups studied between the dose of L-thyroxine and the level of TSH in the blood (*p* = 0.0197). No other statistically significant relationships were observed (*p* < 0.05). Next, multiple (bilateral) comparisons were made to assess any statistically significant differences between subgroups. Significant differences in TSH levels in groups based on the dose of L-thyroxine taken were observed between patients taking a dose ≤ 50 µg (Group I) and patients taking a dose > 100 µg (Group III). Lower TSH values were obtained in patients with higher doses of L-thyroxine. A detailed comparison of TSH levels, depending on the levothyroxine dose taken, is presented in Table 5 and Figure 1.

## 4. Discussion

There are limited data on the effects of levothyroxine dosage on thyroid autoantibody levels and anthropometric measures in patients with Hashimoto’s thyroiditis. This topic might be relevant to clinical practice, particularly given the widespread belief among patients that higher doses of levothyroxine can modulate autoimmune activity. People suffering from hypothyroidism usually require treatment with levothyroxine for the rest of their lives, so therapy should be closely monitored and the dose adjusted to the individual needs of the patient in order to achieve the best possible treatment effect. It is vital to note that the dose of levothyroxine may change with the patient’s age, clinical condition, comorbidities, or medications taken, as these are factors that affect the effectiveness of levothyroxine [16]. The following factors have been identified as influencing the absorption of levothyroxine: gastroesophageal reflux disease, irritable bowel syndrome, food allergies, lactose intolerance, ulcerative colitis, Crohn’s disease, gastritis (especially *Helicobacter pylori* infection and gastritis associated with autoimmune diseases), and giardiasis infection [17,18]. It is also conceivable that dietary supplements (primarily calcium and iron) and intake of foods or beverages high in fiber, iodine, or soy may have a role to play [19]. In patients treated with levothyroxine for hypothyroidism, the most commonly used dose is 1.6 μg/kg of actual body mass. However, significant differences in levothyroxine dosing have been demonstrated in different age groups, different BMI values, and at different stages of menopause [20]. In the search for more accurate alternatives, taking into account ideal body mass, a uniform dosage of levothyroxine has been administered to patients with long-term primary hypothyroidism in different groups and menopausal periods, but with significant differences in BMI. In contrast, dosing based on lean body mass was uniform across different age groups, BMI subgroups, and menopausal status. Thus, an LBM-based dosing of 2.3 µg/kg might be appropriate [20]. In our study of 70 women diagnosed with Hashimoto’s thyroiditis within the last two years, no association was found between levothyroxine dosage (≤50 μg, 50–100 μg, >100 μg) and anti-thyroid antibodies. Another scientific report demonstrated that patients with positive antibodies exhibited a higher requirement for hormone replacement therapy to achieve target TSH levels in comparison to patients with negative antibodies [21]. Furthermore, the antibody titer was found to be positively associated with increased levothyroxine replacement doses in patients with autoimmune thyroiditis [21]. Our study also presented an absence of a significant correlation between anthropometric measurements and levothyroxine dose, indicating that anthropometric variables such as body weight, height, and body fat content were not substantially associated with the selected levothyroxine dose in the women studied. It should be emphasized that the results presented in our study do not contradict the body-weight-adjusted dosing recommended in the guidelines but rather indicate no independent relationship between absolute dose categories and body composition in the patients studied. These results suggest that factors other than levothyroxine dose played a more substantial role, and that any potential dose-dependent effects may have been masked by cumulative influences. Consequently, the higher body weight of women whose thyroid dysfunction may have contributed to the occurrence of overweight and obesity is no less important than other factors that we did not investigate. The absence of a significant correlation between anthropometric measurements and levothyroxine dosage may indicate the need to adjust the dosage based on hormone test results, especially TSH. It should be emphasized that the literature provides evidence of abnormal anthropometric parameters, particularly BMI, in patients with Hashimoto’s thyroiditis. Hypotheses that may confirm a positive correlation between BMI and TSH concern the role of this hormone in preadipocyte differentiation and induced adipogenesis. In addition, authors have demonstrated positive correlations between leptin and TSH, which should also be taken into account in the diagnostic and therapeutic process for patients [22]. There is evidence confirming that women with a BMI ≥ 30 kg/m^2^ are more likely to develop hypothyroidism and test positive for anti-TPO [15]. A study involving 6303 women in early pregnancy also reported a positive correlation between anti-TPO levels and high BMI [15]. In a study conducted in 2021 by Polish scientists, 47 women with Hashimoto’s thyroiditis were found to have significantly higher body weight than 65 healthy women (73.64 kg vs. 64.36 kg, respectively, *p* < 0.0001), as well as a higher body fat content in this group of women (44.7% vs. 13.8%, *p* < 0.001) [23]. In line with previous research findings, we confirmed a high percentage of body fat in patients, which averaged 35.52%. When analyzing the duration of treatment in women with HT (age: 33.5–55 years) and levothyroxine (median dose of levothyroxine: 50 μg), a tendency towards higher anthropometric parameters such as body weight, BMI and fat mass was observed in patients treated with levothyroxine for less than 2 years compared to those who had been treated for over 2 years [24]. Another noteworthy issue is that patients taking levothyroxine for more than 2 years had a significantly smaller thyroid volume and higher anti-TPO levels compared to those treated for less than 2 years. An increase in anti-TPO levels in individuals treated for more than 2 years may indicate more severe inflammation caused by the longer duration of the disease. Some reports suggest that levothyroxine may have an effect on the gradual reduction in thyroid volume, changes in leptin synthesis and activity, and a slight reduction in antibody concentrations that are exclusively identified in patients with overt hypothyroidism. However, these mechanisms are unclear and require further clinical investigation [24]. In addition, it was found that patients with hypothyroidism had elevated ghrelin levels, which decreased after treatment with levothyroxine, and may also affect the anthropometric parameters of patients [25]. This study also demonstrated that in the group taking the lowest dose of levothyroxine (≤50 µg), TSH levels were highest in this group, suggesting that this dose may be less effective in achieving optimal hormonal control in a substantial proportion of the studied patients. The finding that higher doses of levothyroxine are associated with significantly lower TSH levels is expected and reflects standard pharmacodynamic behavior. In our study, the highest dose (>100 µg) was associated with the lowest TSH levels (*p* = 0.026065), but an increase in anti-TPO levels was also observed. This finding may indicate an association with autoimmune activity and potentially more advanced stages of Hashimoto’s thyroiditis [26]. Conversely, the levels of fT3 and fT4 demonstrated relative stability across all groups, suggesting that the conversion of levothyroxine to active thyroid hormones is adequately and comparably accomplished, with minimal inter-group variations. Consequently, the present findings do not support a prominent role of impaired peripheral conversion in the clinical presentation of Hashimoto’s thyroiditis in the studied population [27]. Differences in TSH levels between groups indicate the need for an individual approach to levothyroxine dosing, especially in the context of thyroid function control and possible excessive autoimmune stimulation [28].

The present study has several limitations that should be acknowledged. The relationships identified should not be interpreted as definitive evidence, and the findings require cautious interpretation. The absence of a normalized levothyroxine dose tailored to body weight or lean mass limits interpretability and may partially explain the lack of association with anthropometric variables. Furthermore, the results reflect associations observed at a single time point and do not capture the long-term effects of escalating the levothyroxine dose.

An additional limitation is the unequal and limited sample size following stratification by levothyroxine dosage (group I: 30 patients; group II: 30 patients; group III: 10 patients). In particular, the small number of participants in group III substantially reduces statistical power and limits the robustness of between-group comparisons. Moreover, anti-TG antibody levels demonstrated pronounced variability, with standard deviations exceeding mean values, especially in the smaller subgroups, further complicating interpretation and increasing the risk.

Taken together, these limitations indicate that the observed lack of association between levothyroxine dose and anti-TPO or anti-TG antibody levels should be interpreted with caution. Larger, longitudinal studies with balanced group sizes and improved control of antibody variability are warranted to clarify the relationship between levothyroxine dosage, thyroid autoimmunity, and anthropometric parameters in patients with Hashimoto’s thyroiditis and to support the development of more individualized therapeutic strategies.

## 5. Conclusions

The findings of our studies indicate that adjusting the levothyroxine dose is imperative to stabilize TSH, fT3, and fT4 levels. It is conceivable that elevated levothyroxine doses may result in diminished levels of anti-TPO and anti-TG antibodies; however, the present studies did not demonstrate a substantial correlation. The levothyroxine dose, in terms of absolute daily dose, is not independently associated with thyroid autoantibody levels in women with relatively recent HT, and the regulation of antibody levels reflects the immune processes triggered by the disease.

## Figures and Tables

**Table 1:** Mean of selected anthropometric and biochemical parameters of the study group (n = 70).

Parameters	Avg	SD
Age (years)	36.68	8.34
Height (cm)	166.19	5.74
Body weight (kg)	71.54	13.03
Body fat (%)	35.52	6.75
Anti-TPO (0–34 IU/mL)	232.83	276.98
Anti-TG (0–115 IU/mL]	335.85	615.92
TSH (0.270–4.200 μIU/mL)	2.91	2.35
fT3 (2.00–4.40 pg/mL)	2.97	0.43
fT4 (0.93–1.70 ng/dL)	1.29	0.20
Avg dose of L-thyroxine (μq/day)	71.16	36.15

Avg—average; SD—standard deviation; anti-TPO—anti-thyroid peroxidase; anti-TG—anti-thyroglobulin antibodies; TSH—thyroid-stimulating hormone; fT3—free triiodothyronine; fT4—free thyroxine; n—number of participants.

**Table 2:** Correlation of L-thyroxine dose with anthropometric measurement (n = 70).

Pair of Variables	Spearman Rank Order Correlation and *p*-Value
L-thyroxine dose [μg] & age [years]	*p* = 0.466793
L-thyroxine dose [μg] & height [cm]	*p* = 0.508984
L-thyroxine dose [μg] & body mass [kg]	*p* = 0.178705
L-thyroxine dose [μg] & body fat content [%]	*p* = 0.432847

*p*—*p*-value < 0.05; n—number of participants.

**Table 3:** Comparison of mean biochemical parameters and differences in biochemical measurements in subgroups based on L-thyroxine dosage.

	Anti-TPO (IU/mL)	Anti-TG (IU/mL)	TSH (μIU/mL)	fT3 (pg/mL)	fT4 (ng/dL)
I Group (Avg and SD; n = 30)	231.17 ± 213.32	309.99 ± 700.77	3.10 ± 1.47	3.03 ± 0.39	1.26 ± 1.18
II Group (Avg and SD; n = 30)	170.93 ± 205.57	393.24 ± 612.95	3.12 ± 3.09	2.93 ± 0.46	1.32 ± 0.21
III Group (Avg and SD; n = 10)	423.51 ± 475.23	247.00 ± 187.72	1.68 ± 1.30	2.92 ± 0.42	1.34 ± 0.19
I vs. II	*p* = 0.152	*p* = 0.329	*p* = 0.193	*p* = 0.438	*p* = 0.176
II vs. III	*p* = 0.129	*p* = 0.708	*p* = 0.563	*p* = 0.963	*p* = 0.864
I vs. III	*p* = 0.223	*p* = 0.492	*p* = 0.026	*p* = 0.639	*p* = 0.492

anti-TPO—anti-thyroid peroxidase; anti-TG—anti-thyroglobulin antibodies; TSH—thyroid-stimulating hormone; fT3—free triiodothyronine; fT4—free thyroxine; I group: ≤50 μg L-thyroxine; II group: 50–100 μg L-thyroxine; III group: >100 μg L-thyroxine; Avg—average; SD—standard deviation; n—number of participants; *p*—*p*-value < 0.05.

**Table 4:** Analysis of variance of levothyroxine dose with anti-TPO, anti-TG, and thyroid hormone profile value—Kruskal–Wallis rank ANOVA.

Dependent Variable	Kruskal–Wallis Statistic and *p*-Value	
Anti-TPO (0–34 IU/mL)	H = 5.383984	*p* = 0.0677
Anti-TG (0–115 IU/mL)	H = 1.707592	*p* = 0.4258
TSH (0.270–4.200 μIU/mL)	H = 7.856646	*p* = 0.0197
fT3 (2.00–4.40 pg/mL)	H = 0.6455844	*p* = 0.7241
fT4 (0.93–1.70 ng/dL)	H = 1.861525	*p* = 0.3943

Anti-TPO—anti-thyroid peroxidase; anti-TG—anti-thyroglobulin antibodies; TSH—thyroid-stimulating hormone; fT3—free triiodothyronine; fT4—free thyroxine; H—test statistic for the Kruskal–Wallis test; *p*—*p*-value < 0.05.

**Table 5:** Comparison of TSH levels depending on the levothyroxine dose taken—multiple comparisons (bilateral).

Levothyroxine Dose [μg]	*p* Value for Multiple Comparisons (Bilateral); TSH [0.270–4.200 μIU/mL] Independent (Grouping) Variable: Thyroxine Groups Kruskal–Wallis Statistic: H (2, N = 70) = 7.856646 *p* = 0.0197		
	≤50 R:42.450	>50 ≤ 100 R:32.733	>100 R:22.950
Group I ≤ 50		*p* = 0.193303	*p* = 0.026065
Group II > 50 ≤ 100	*p* = 0.193303		*p* = 0.563991
Group III > 100	*p* = 0.026065	*p* = 0.563991	

## Data Availability

The data presented in this study are available on request from the corresponding author.

## Abbreviations

The following abbreviations are used in this manuscript:

anti-TPO	thyroid peroxidase antibodies
anti-TG	thyroglobulin antibodies
HT	Hashimoto’s thyroiditis
fT3	free triiodothyronine
fT4	free thyroxine
TSH	thyroid-stimulating hormone

## References

[1] Luty J., Ruckemann-Dziurdzińska K., Witkowski J.M., Bryl E. (2019). Immunological aspects of autoimmune thyroid disease—Complex interplay between cells and cytokines. Cytokine.

[2] Ralli M., Angeletti D., Fiore M., D’AGuanno V., Lambiase A., Artico M., de Vincentiis M., Greco A. (2020). Hashimoto’s thyroiditis: An update on pathogenic mechanisms, diagnostic protocols, therapeutic strategies, and potential malignant transformation. Autoimmun. Rev..

[3] Jin B., Wang S., Fan Z. (2022). Pathogenesis Markers of Hashimoto’s Disease—A Mini Review. FBL.

[4] Vargas-Uricoechea H. (2023). Molecular Mechanisms in Autoimmune Thyroid Disease. Cells.

[5] Pyzik A., Grywalska E., Matyjaszek-Matuszek B., Roliński J. (2015). Immune disorders in Hashimoto’s thyroiditis: What do we know so far?. J. Immunol. Res..

[6] Shen X., Yan X., Xie B., Xu D., Wang K., Zhu J., Li J., Zhang X., Cao F. (2015). Genetic variants of interleukin-4 gene in autoimmune thyroid diseases: An updated meta-analysis. Autoimmunity.

[7] Lee H.J., Li C.W., Hammerstad S.S., Stefan M., Tomer Y. (2015). Immunogenetics of Autoimmune Thyroid Diseases: A Comprehensive Review. J. Autoimmun..

[8] Schmidt M., Voell M., Rahlff I., Dietlein M., Kobe C., Faust M., Schicha H. (2008). Long-term follow-up of antithyroid peroxidase antibodies in patients with chronic autoimmune thyroiditis (Hashimoto’s thyroiditis) treated with levothyroxine. Thyroid.

[9] Stempler M., Bakos B., Solymosi T., Kiss A., Ármós R.L., Szili B., Mészáros S., Tőke J., Szűcs N., Reismann P. (2024). Analysis of factors influencing the dose of levothyroxine treatment in adequately controlled hypothyroid patients of different etiologies. Heliyon.

[10] Altun R., Canpolat A.G., Demir Ö., Erdogan M.F. (2021). The course of Autoimmune Thyroiditis in WOMEN. Acta Endocrinol..

[11] Mammen J.S.R., Cappola A.R. (2021). Autoimmune Thyroid Disease in Women. JAMA.

[12] Rozwandowicz A.M., Stuss M., Michalska-Kasiczak M., Sewerynek E. (2024). Body composition analysis in patients with Hashimoto’s disease and vitamin D defi ciency. J. Med. Sci..

[13] Tomov D.G., Levterova B.A., Troev D.M., Miteva M.Z., Mihaylova V.N., Uzunova Y.I., Divarova V.V., Orbetzova M.M. (2023). Serum levels of leptin and adiponectin in patients with autoimmune Hashimotos thyroiditis. Folia Med..

[14] Marzullo P., Minocci A., Tagliaferri M.A., Guzzaloni G., Di Blasio A., De Medici C., Aimaretti G., Liuzzi A. (2010). Investigations of thyroid hormones and antibodies in obesity: Leptin levels are associated with thyroid autoimmunity independent of bioanthropometric, hormonal, and weight-related determinants. J. Clin. Endocrinol. Metab..

[15] Han C., Li C., Mao J., Wang W., Xie X., Zhou W., Li C., Xu B., Bi L., Meng T. (2015). High Body Mass Index Is an Indicator of Maternal Hypothyroidism, Hypothyroxinemia, and Thyroid-Peroxidase Antibody Positivity during Early Pregnancy. Biomed. Res. Int..

[16] Duntas L.H., Jonklaas J. (2019). Levothyroxine Dose Adjustment to Optimise Therapy Throughout a Patients Lifetime. Adv. Ther..

[17] Caron P., Grunenwald S., Persani L., Borson-Chazot F., Leroy R., Duntas L. (2022). Factors influencing the levothyroxine dose in the hormone replacement therapy of primary hypothyroidism in adults. Rev. Endocr. Metab. Disord..

[18] Virili C., Brusca N., Capriello S., Centanni M. (2021). Levothyroxine Therapy in Gastric Malabsorptive Disorders. Front. Endocrinol..

[19] McMillan M., Rotenberg K.S., Vora K., Sterman A.B., Thevathasan L., Ryan M.F., Mehra M., Sandulli W. (2016). Comorbidities, Concomitant Medications, and Diet as Factors Affecting Levothyroxine Therapy: Results of the CONTROL Surveillance Project. Drugs R D.

[20] Venkateswarlu D., Goroshi M., Ganakumar V., Ghatnatti V., Kotla S. (2025). Lean Body Mass as a Predictor of Levothyroxine Requirement in Primary Hypothyroidism as Compared to Actual Body Weight. J. Endocr. Soc..

[21] Okuroglu N.N., Ozdemir A., Sertbas Y., Sancak S. (2017). The relationship between thyroid antibody titer and levothyroxine dose in patients with overt primary hypothyroidism. Ann. Saudi Med..

[22] Mousa U., Bozkuş Y., Kut A., Demir C.C., Tutuncu N.B. (2018). Fat distribution and metabolic profile in subjects with hashimotos thyroiditis. Acta Endocrinol..

[23] Malczyk E., Wyka J., Malczyk A. (2021). Body composition and Hashimoto disease. Rocz. Panstw. Zakl. Hig..

[24] Popławska-Kita A., Siewko K., Telejko B., Kościuszko-Zdrodowska M., Hryniewicka J., Szelachowska M., Milewski R., Górska M. (2014). Body mass analysis in patients with Hashimoto thyroiditis. Prog. Health Sci..

[25] Kim K.J., Kim B.Y., Mok J.O., Kim C.H., Kang S.K., Jung C.H. (2015). Serum Concentrations of Ghrelin and Leptin according to Thyroid Hormone Condition, and Their Correlations with Insulin Resistance. Endocrinol. Metab..

[26] Szczuko M., Syrenicz A., Szymkowiak K., Przybylska A., Szczuko U., Pobłocki J., Kulpa D. (2022). Doubtful Justification of the Gluten-Free Diet in the Course of Hashimotos Disease. Nutrients.

[27] Batóg G., Dołoto A., Bąk E., Piątkowska-Chmiel I., Krawiec P., Pac-Kożuchowska E., Herbet M. (2023). The interplay of oxidative stress and immune dysfunction in Hashimoto’s thyroiditis and polycystic ovary syndrome: A comprehensive review. Front. Immunol..

[28] Van Uytfanghe K., Ehrenkranz J., Halsall D., Hoff K., Loh T.P., Spencer C.A., Köhrle J. (2023). Thyroid Stimulating Hormone and Thyroid Hormones (Triiodothyronine and Thyroxine): An American Thyroid Association-Commissioned Review of Current Clinical and Laboratory Status. Thyroid.

